# Dietary habits of pregnant women in Bulgaria

**DOI:** 10.1093/eurpub/ckac130.242

**Published:** 2022-10-25

**Authors:** R Chamova, M Panteleeva

**Affiliations:** 1 Department of Hygiene and Epidemiology, Medical University of Varna, Varna, Bulgaria; 2 Department of Disaster Medicine and Maritime Medicine, Medical University of Varna, Varna, Bulgaria

## Abstract

**Background:**

The optimal nutritional status of the mother is one of the most influential non-genetic factors for the healthy development of the fetus. In recent years, more and more scientific evidence has been accumulating that her dietary habits and nutritional status determine the fetal development and the health of the offspring.

**Methods:**

A cross - sectional study of pregnant women's dietary habits was conducted online. A questionnaire is attached, including questions about the diet, the frequency of consumption of certain food groups, application of alternative eating patterns and more. The analysis of the results is done with a software statistical package Jamovi ver. 2.3.0.

**Results:**

The servey is conducted among 117 women with a mean age of 30.4 ± 4.88 years. The majority of them have changed their dietary habits after registering a pregnancy (72.6%). Among all respondents, 18.8% haven't got a fixed diet. 67% of the respondents eat 3 - 4 times a day, and 5.1% - less than three times a day. The relative share of pregnant women who consume milk and dairy products every day is 41% and 47%, respectively. None of the respondents restrains from consuming dairy products. Only 5.1% of the respondents do not consume milk. Six of the surveyed women (5.1%) do not eat meat and 14 (12%) do not eat fish. The relative share of women who eat fish 1 - 2 times a week is 29.9%. The majority of women (88.9%) doesn't consume alcohol during pregnancy. The relative share of vegetarians is 6%. There are no vegans among the women surveyed.

**Conclusions:**

The majority of pregnant women surveyed follow the recommendations for healthy eating. There are women at risk of developing nutritional deficiencies among the respondents - macro - and micronutrients, which is a threat for maternal health, the course of pregnancy, as well as the growth and development of offspring.

**Key messages:**

- The key to a normal pregnancy and primary prevention of complications is to establish healthy dietary habits.
- Malnutrition, overfeeding and unbalanced nutrition during pregnancy can adversely affect the offspring health at all stages of its live.

